# Concurrent alcohol and smoking use among American Indian and Alaska Native adults participating in a contingency management clinical trial for alcohol use disorder

**DOI:** 10.3389/fpubh.2026.1749377

**Published:** 2026-03-11

**Authors:** Kelley J. Jansen, Megan Puzia, Jalene L. Herron, Abram J. Lyons, Michael McDonell, Katherine Hirchak, The HONOR Study Team

**Affiliations:** 1 Department of Psychology, University of Alaska Anchorage, Anchorage, AK, United States; 2 Department of Community and Behavioral Health, Elson S. Floyd College of Medicine, Washington State University, Spokane, WA, United States; 3 Promoting Research Initiatives in Substance Use and Mental Health Collaborative, Washington State University, Spokane, WA, United States; 4 Department of Psychology, University of New Mexico, Albuquerque, NM, United States; 5 College of Education & Human Development, Social Work Program, University of Delaware, Newark, DE, United States

**Keywords:** Alaska Native and American Indian, concurrent use, contingency management, smoking, tobacco cessation

## Abstract

**Introduction:**

The co-use of alcohol and cigarettes is common and associated with poor health outcomes. However, little is known about the prevalence of cigarette smoking among American Indian and Alaska Native (AI/AN) adults seeking treatment for alcohol use disorder (AUD), whether participation in AUD treatment is associated with changes in cigarette smoking or patterns of concurrent alcohol and cigarette use in this population.

**Methods:**

This study reports a secondary analysis of concurrent alcohol and cigarette use among AI/AN participants in a randomized controlled trial of contingency management (CM) for AUD (*N* = 158). Participants completed Timeline Followback assessments of daily cigarette use and alcohol consumption for the 30 days before baseline and throughout the 20-week study period. Generalized linear mixed models were used to evaluate changes in smoking over time and associations between alcohol use, accounting for study period, repeated measures, and recent use behaviors.

**Results:**

Baseline assessments indicated a high prevalence of cigarette smoking (66.7%, *n* = 96/144). Across the study period, participants in both conditions demonstrated reductions in cigarette smoking, particularly during the post-baseline phase (*B* = −0.54; *p* < 0.01). No statistically significant differences in smoking outcomes were observed between the CM and control conditions. After controlling for recent use patterns, a significant positive association was observed between daily alcohol consumption and same-day cigarette smoking (*B* = 0.12; *p* < 0.01).

**Discussion:**

Two-thirds of AI/AN adults with AUD in this sample also smoked cigarettes. Reductions in smoking were observed over time across both study conditions, including during the pre-randomization observation phase, suggesting that changes were not attributable to the CM intervention. Daily alcohol consumption and cigarette smoking were positively correlated, indicating concurrent substance use. Given these findings, smoking cessation interventions should be provided to AI/AN adults.

## 1 Introduction

Despite decades of prevention and cessation programs, smoking remains one of the leading causes of preventable death and disease in the United States [US; (1)]. Cigarette use also negatively affects American Indian and Alaska Native (AI/AN) peoples. On average, a higher proportion of AI/AN peoples smoke cigarettes (20.9%) compared to their non-AI/AN counterparts [14.0%; (2)]. Although the number of cigarettes smoked per day on average is lower for AI/AN peoples than for non-Hispanic White individuals, AI/AN peoples experience a greater burden of commercial tobacco–related illness (3–5).

The concurrent use of alcohol and cigarettes reflects broader systemic and structural inequities that disproportionately affect AI/AN communities, including limited access to culturally grounded cessation resources, historical trauma, and discrimination within healthcare systems (6–9). Commercial tobacco is distinct from the tobacco used in cultural and traditional practices in some Indigenous communities. Commercial tobacco is mass-produced, widely marketed, generates substantial profits, and contains harmful chemicals that are not present in traditional tobacco (10, 11, 44). In the original trial, traditional tobacco use was not assessed; therefore, this study focuses exclusively on the smoking of commercially manufactured cigarettes. Traditional tobacco use is not included, nor are other products or modes of smoking, such as electronic cigarettes or cannabis.

The health risks associated with alcohol and cigarette use are well known, and concurrent use further increases the risk of adverse outcomes, including cancer, asthma, hypertension, diabetes, and mental health conditions (1, 12, 13). In addition, individuals with an alcohol use disorder (AUD) are three times more likely to smoke cigarettes than those without AUD (2, 14, 16, 45–48). Alcohol and cigarette co-use among AI/AN peoples has not been well characterized in the current literature, particularly among AI/AN peoples in AUD treatment-seeking settings. Although it is well known that alcohol and cigarettes are commonly used together, existing studies among AI/AN peoples have largely been epidemiological and based on national or regional samples, limiting the ability to examine concurrent alcohol and cigarette use within the same treatment-seeking sample (17). Consequently, the current literature is limited regarding concurrent alcohol and cigarette use in the context of treatment-seeking AI/AN adults receiving treatment for AUD.

Contingency management (CM) is an AUD treatment that provides incentives for abstinence from alcohol, as confirmed by biological samples such as urine, blood, and breath tests (18–20). Although CM is a Western treatment intervention, its cultural acceptability has been examined and found to be adaptable and aligned with AI/AN values through the tailoring of incentives, methods of delivery, and treatment locations (21, 22, 49).

Two clinical trials of CM conducted in AI/AN communities demonstrated reductions in alcohol and stimulant use (21, 22, 50, 51). In one trial reinforcing alcohol use only, secondary reductions in cannabis use were also observed (21). Another study of CM for AUD in non-AI/AN adults found that participants who received CM were three times more likely to submit a smoking-negative breath sample compared to control participants (23). Other studies have reported secondary reductions in smoking during CM interventions targeting stimulant use (24, 25). Although these findings suggest a secondary effect of CM on smoking, this effect appears to depend on whether smoking is directly targeted within the intervention.

Given the limited evidence on cigarette use among AI/AN adults seeking treatment for AUD, and the literature on the secondary impact of CM for AUD on cigarette use in non-AI/AN samples, we sought to document the rate of cigarette smoking in AI/AN AUD treatment-seeking sample recruited from three diverse AI/AN communities, assess the secondary impact (i.e., changes in behavior not directly targeted by the CM intervention) of CM on cigarette use within this sample, and characterize the relationship between alcohol use and cigarette smoking using intensive longitudinal data.

## 2 Methods

### 2.1 Study sample

Participant inclusion criteria were as follows: (1) AI/AN peoples aged 18 years or older; (2) reporting one or more days of heavy drinking is based on the NIAA criteria for 5+/day for men and 4+/day for women in the past 30 days; and (3) meeting DSM-IV criteria for current alcohol dependence. DSM-IV alcohol dependence was assessed using the Mini International Neuropsychiatric Interview questionnaire [MINI; (26)].

Exclusion criteria were as follows: (1) reporting more days of illicit drug use than alcohol use during the past 90 days; (2) experiencing alcohol withdrawal symptoms in the past 12 months; and (3) having medical or psychiatric conditions that could jeopardize safe participation in the study.

### 2.2 Procedures

Alcohol use was measured using ethyl glucuronide (EtG), an alcohol biomarker produced by the liver and excreted in urine. EtG can be used to detect heavy alcohol use for up to 5 days and lighter alcohol use for up to 3 days (27). A benchtop analyzer located at each of the three study community sites was used to analyze urine samples for EtG levels (Indiko Benchtop Analyzer; ThermoFischer Scientific) using an enzyme immunoassay (Diagnostic Reagents).

Eligible participants completed a 4-week observation phase before randomization, which included attendance at study visits twice weekly. Observation periods have been previously implemented to increase retention and assist participants in becoming familiar with study procedures (23, 28). Following the observation phase, participants who met randomization criteria (i.e., submission of 50% or more urine samples and at least one alcohol-positive urine test during the 4-week pre-randomization observation period) were randomized to either the CM group or control group.

Following randomization, participants completed 12 weeks of twice-weekly study visits lasting approximately 15 min. Participants assigned to the CM condition received the CM intervention for alcohol abstinence (EtG < 150 ng/mL), whereas participants in the control condition received reinforcers for attending study visits regardless of alcohol use. After the 12-week intervention period, follow-up visits were completed at months 1, 2, and 3.

The reinforcement schedule used in this study was a variable magnitude of reinforcement procedure. All participants drew five chips from a bowl containing 500 chips. Fifty percent of the chips included words such as “good job!” in each community’s respective Tribal language(s). An additional 41.8% of the chips indicated a “small reward,” which could be exchanged for an item worth approximately one US dollar. Eight percent of the chips indicated a “large reward,” which could be exchanged for an item worth approximately US$20. One chip (0.2%) indicated a “jumbo reward,” which could be exchanged for an item worth approximately US$80. Although the reinforcers were identical across conditions, participants randomized to the CM group received increasing chances of receiving a reinforcer for alcohol abstinence as the number of alcohol-negative urinalyses increased. Drug test results from urinalyses were not incorporated into the reinforcement schedule; therefore, participants received reinforcers regardless of drug testing results. Participants were followed once per month for 3 more months post-intervention. Retention in the original study was low, which is common among treatment-seeking populations due to multiple barriers to care (29).

### 2.3 Measures

To assess cigarette smoking and nicotine dependence at baseline, the Fagerstrom Test for Nicotine Dependence (FTND) was administered (30, 31). The FTND is a self-report instrument that measures an individual’s level of physical dependence on nicotine. It consists of six items that evaluate the quantity of cigarette consumption, the compulsion to use, and dependence.

Participants also self-reported cigarette use on each day using the Timeline Followback (TLFB) method (32). The TLFB is a validated method for obtaining quantitative estimates of alcohol and cigarette use to monitor and evaluate changes over time. It can be administered by an interviewer or self-administered using paper or electronic formats. In this study, a research assistant recorded participants’ number self-reported number of cigarettes smoked on a printed calendar twice weekly. At baseline, participants reported cigarette use for the 30 days before study entry; during the study, participants reported cigarette use for the three to five days preceding each study visit.

### 2.4 Statistical analysis

Analyses were conducted using SPSS [version 29; (33)]. Baseline smoking status was determined based on responses to the FTND entry question, “Do you smoke tobacco for non-ceremonial reasons?” Only participants who responded affirmatively to this item were included in the analytic sample.

Descriptive statistics were used to characterize the sample in terms of demographics, comorbidities, alcohol use, and smoking behaviors. Chi-square tests and *t*-tests were used to assess group differences in baseline smoking behaviors. Predictive analyses utilized generalized linear mixed regression models (GLMMs) to account for repeated observations, variability in measurement timing, and missing-data patterns across participants (34). All models used restricted maximum likelihood estimation and specified an autoregressive covariance structure. All tests were two-sided, and a *p*-value of <0.05 was considered statistically significant.

To examine changes in smoking over time, we regressed self-reported cigarettes smoked (from TLFB data) were regressed onto a predictor set that included study phase (induction and post-randomization periods, relative to the pre-study baseline period), post-randomization study group (treatment as usual [TAU] = 0 vs. CM = 1), and a time-by-phase (pre- vs. post-randomization) interaction. To account for differences in weekday and weekend substance use [e.g., (35–37)], models assessing changes in smoking over time used the number of cigarettes smoked per day aggregated across one-week periods (20 total weeks). Models additionally controlled for the average number of daily cigarettes smoked and daily alcoholic drinks during the pre-study period.

To examine the concurrency of smoking and alcohol use over time, models regressed daily-level self-reported alcohol consumption (number of standard alcoholic drinks per day) onto same-day cigarette smoking (number of cigarettes). To control for the influence of recent alcohol and cigarette use, models included lagged variables calculated as rolling averages of daily alcoholic drinks consumed and daily cigarettes smoked during the previous seven days.

## 3 Results

At baseline, two-thirds of the sample reported smoking cigarettes for non-ceremonial reasons (66.7%, *n* = 96/144). Sample demographics are presented in Table 1. In the 30 days before enrollment, participants smoked an average of 7.4 cigarettes per day (*SD* = 5.6; i.e., 51.2 cigarettes per week, *SD* = 39.4) and consumed an average of 5.3 alcoholic drinks per day (*SD* = 4.5; i.e., 37.7 drinks per week, *SD* = 31.6). Most participants had low (53.5%, *n* = 46) to moderate (39.5%, *n* = 34) nicotine dependence score (mean Fagerstrom = 3.26, *SD* = 2.47). There were no significant group differences at baseline in smoking rates (*χ*^2^ = 0.50, *p* = 0.48), Fagerstrom scores (*t* = 1.64, *p* = 0.10), average daily number of cigarettes smoked (*t* = 1.70, *p* = 0.09), or average daily alcohol consumption (*t* = −1.50, *p* = 0.14).

**Table 1:** Demographic characteristics of the sample.

Characteristic	TAU (*N* = 78)		CM (*N* = 66)		Total sample (*N* = 144)	
	*n*	%	*n*	%	*n*	%
Gender						
Male	37	47.4	40	60.6	77	53.5
Female	40	51.3	25	37.9	65	45.1
Other	1	1.3	1	1.5	2	1.4
Housing status						
Stably housed	44	56.4	42	63.6	86	59.7
Temporary housing	5	6.4	7	10.6	12	8.3
Homeless	12	15.4	10	15.2	22	15.3
Other	17	21.8	7	10.6	24	16.7
Living on reservation	28	35.9	22	33.3	50	34.7
Employment						
Full-time	19	24.4	24	36.3	43	29.9
Part-time	26	33.3	24	36.4	50	34.7
Unemployed	24	30.8	12	18.2	36	25.0
Other	9	11.5	6	9.1	15	10.4
Age (*M*, *SD*)	41.7	11.7	43.2	11.1	42.4	11.4
Education (*M* years, *SD*)	12.1	2.2	12.8	2.1	12.4	2.1

CM, contingency management; TAU, treatment as usual.

**Figure 1:**
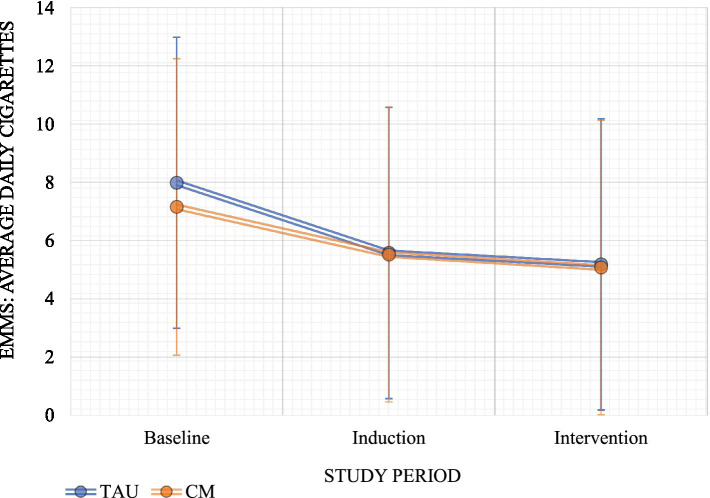
Estimated marginal means: average cigarettes by study period and treatment group. EMM, estimated marginal means.

The results from models assessing changes in weekly cigarette smoking are presented in Table 2. The models demonstrated significant main effects of the study period, in which compared to baseline, cigarette smoking decreased during both the induction and post-randomization phases of the study (Figure 1). The main effect of group and the group-by-period interaction was not statistically significant, indicating that CM did not have secondary effects on cigarette smoking.

**Table 2:** Generalized linear mixed model of secondary effects of CM on cigarette smoking.

Model term	Coefficient	*SE*	*t*	*p*	95% CI	
					LL	UL
Intercept	2.54	2.59	0.98	0.33	−2.53	7.61
Post-baseline period	−2.08	0.23	−8.90	<0.001	−5.54	−1.62
Post-randomization period	−0.46	0.21	−2.21	0.03	−0.87	−0.05
Avg daily cigarettes pre-study	0.12	0.02	5.41	<0.001	0.07	0.16
Group = CM	−0.23	0.24	−0.93	0.35	−0.71	0.25
Group*post-randomization	0.11	0.31	0.35	0.73	−0.50	0.72

CM, contingency management.

**Figure 2:**
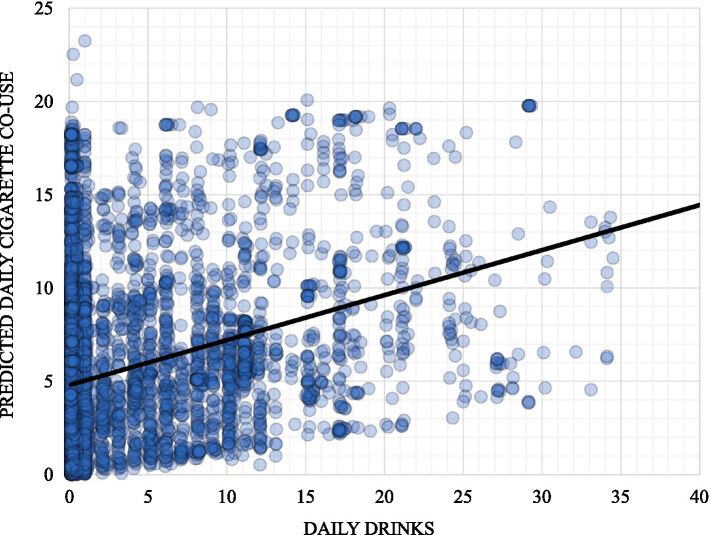
Predicted daily cigarette co-use by same-day daily drinks.

Models examining concurrent substance use indicated that, after controlling for participants’ pre-study and recent alcohol use and cigarette smoking behaviors (i.e., past-week rolling averages), the association between the number of alcoholic drinks consumed and the number of cigarettes smoked remained statistically significant at the daily level (see Table 3; Figure 2).

**Table 3:** Generalized linear mixed model of daily alcohol consumption predicting same-day cigarette smoking.

Model term	Coefficient	*SE*	*t*	*p*	95% CI	
					LL	UL
Intercept	0.72	2.15	0.33	0.74	−3.50	4.93
Post-baseline period	−0.54	0.19	−2.91	0.004	−0.90	−0.18
Post-randomization period	0.09	0.11	0.79	0.43	−0.13	0.30
Avg daily cigarettes pre-study	0.11	0.01	8.29	<0.001	0.08	0.13
Avg daily drinks pre-study	−0.03	0.01	−2.34	0.02	−0.06	−0.01
Avg cigarettes in past 7 days	0.79	0.01	53.21	<0.001	0.76	0.82
Avg drinks in past 7 days	−0.06	0.01	−4.51	<0.001	−0.09	−0.04
Same-day drinks	0.12	0.01	19.84	<0.001	0.11	0.13

## 4 Discussion

This study was a secondary data analysis of concurrent alcohol and cigarette use among AI/AN participants enrolled in a randomized controlled trial of CM for AUD. The study was conducted in partnership with three AI/AN communities located in Alaska, the Northern Plains, and the Northwest. A key finding was the high prevalence of cigarette smoking among AI/AN adults with AUD, with approximately two-thirds of participants reporting current smoking. This prevalence is notably higher than the national average and suggests that incorporating smoking cessation interventions into AUD treatment could be beneficial.

Previous CM studies have reported secondary reductions in smoking among participants receiving CM for AUD or illicit drug use (38–41); however, we did not observe a comparable secondary reduction in cigarette smoking in the present study. Consistent with this, CM for alcohol use did not demonstrate secondary effects on cigarette smoking, likely because smoking was not directly targeted within this intervention. Nevertheless, participants in both the CM and control conditions showed decreases in the average number of cigarettes smoked per week over the course of the study.

Within this AI/AN treatment-seeking sample, we observed a significant same-day association between alcohol consumption and cigarette smoking. Participants demonstrated reductions in smoking and drinking during the first 4 weeks of the study, before randomization into the treatment phase. This pattern suggests that treatment engagement or motivation associated with seeking treatment may contribute to reduction of cigarette smoking among AI/AN adults, rather than effects attributable to the CM intervention itself. Previous research among non-AI/AN adults has similarly shown that reductions in smoking during the first year of treatment are associated with long-term reductions in alcohol dependence (42).

The results highlight the potential value of integrating alcohol and smoking cessation treatments and may help inform policymakers and Tribal and healthcare organization leaders regarding feasible and effective strategies to support smoking reduction or cessation. It is possible that CM-based approaches could be offered within AUD treatment as an optional service for individuals interested in reducing cigarette smoking. CM may also be adaptable to primary care settings and could serve as an alternative to, or in conjunction with, medication-assisted treatments for smoking cessation (43). Future research may benefit from using ecological momentary assessment or similar approaches to capture real-time co-use of alcohol and cigarettes among AI/AN adults and to better characterize patterns of co-use of these substances and the contexts in which these substances are used.

### 4.1 Limitations

This study has several limitations. First, the clinical trial from which these data were drawn did not use carbon monoxide breath testing, cigarette smoking was assessed solely via self-report to estimate participants’ smoking throughout the study. Second, although EtG was collected as part of the original trial, urine EtG was not analyzed to measure alcohol use in the present analysis.

Third, the findings may not be generalizable to all AI/AN communities because data were collected from only three communities. However, a strength of the study is that these communities included both rural and urban populations. Fourth, although common in treatment-seeking populations, the clinical trial experienced relatively low retention, resulting in a smaller analytic sample. This may have limited the ability to detect longitudinal effects of CM for AUD on cigarette smoking.

Finally, since the original grant was developed before the release of the *Diagnostic and Statistical Manual 5th Edition* (DSM-5), the study’s inclusion criteria for AUD were based on DSM-IV criteria for alcohol dependence rather than current DSM-5 criteria. This may limit the generalizability of findings to studies using DSM-5 definitions and should be considered in future replications.

## 5 Conclusion

Smoking cessation interventions have been a central component of public and behavioral health efforts for decades; however, AI/AN peoples continue to have the highest rates of cigarette smoking compared to other racial and ethnic groups and are disproportionately affected by smoking-related health complications. In addition, this study corroborates existing literature demonstrating the high co-occurrence of alcohol and cigarette use.

As institutional and systemic barriers affect help-seeking among individuals who wish to quit smoking, the implementation of novel interventions are needed. Integrating or delivering parallel cigarette smoking interventions within alcohol treatment represents an important step toward improving the health and well-being of AI/AN individuals, families, and communities.

## Data Availability

The datasets presented in this article are not readily available because data are owned by the participating Tribes and are not publicly available. Requests to access the datasets should be directed to mmcdonell@wsu.edu.
